# Predictors of successful endovascular recanalization in patients with symptomatic nonacute intracranial large artery occlusion

**DOI:** 10.1186/s12883-023-03424-y

**Published:** 2023-10-19

**Authors:** Shuo Yan, Hao Feng, Lin Ma, Ji-Chong Xu, Hong-Jie Han, Hong-En Huang, Hua-Qiao Tan, Chun Fang

**Affiliations:** Department of Interventional Radiology, Tongji Hospital, Tongji University School of Medicine, No. 389, Xin Chun Road, Shanghai, 200065 China

**Keywords:** Endovascular recanalization, Intracranial artery, Nonacute occlusion, Angioplasty, Stenting

## Abstract

**Background:**

Endovascular recanalization in patients with symptomatic nonacute intracranial large artery occlusion (ILAO) has been reported to be feasible, but technically challenging. This study aimed to determine the predictors of successful endovascular recanalization in patients with symptomatic nonacute ILAO.

**Methods:**

The outcomes of endovascular recanalization attempts performed in 70 consecutive patients showing symptomatic nonacute ILAO with hemodynamic cerebral ischemia between January 2016 to December 2022 were reviewed. Potential variables, including clinical and radiological characteristics related to technical success, were collected. Univariate analysis and multivariate logistic regression were performed to identify predictors of successful recanalization for nonacute ILAO.

**Results:**

Technically successful recanalization was achieved in 57 patients (81.4%). The periprocedural complication rate was 21.4% (15 of 70), and the overall 30-day morbidity and mortality rates were 7.1% (5 of 70) and 2.9% (2 of 70), respectively. Univariate analysis showed that successful recanalization was associated with occlusion duration, stump morphology, occlusion length, slow distal antegrade flow sign, and the presence of bridging collateral vessels. Multivariate analysis showed that occlusion duration ≤ 3 months (odds ratio [OR]: 22.529; 95% confidence interval [CI]: 1.636-310.141), tapered stump (OR: 7.498; 95% CI: 1.533–36.671), and occlusion length < 10 mm (OR: 7.049; 95% CI: 1.402–35.441) were independent predictive factors for technical success of recanalization.

**Conclusions:**

Occlusion duration ≤ 3 months, tapered stump, and occlusion length < 10 mm were independent positive predictors of technical success of endovascular recanalization for symptomatic nonacute ILAO. These findings may help predict the likelihood of successful recanalization in patients with symptomatic nonacute ILAO and also provide a reference for the selection of appropriate patients. Further prospective and multicenter studies are required to validate our findings.

## Introduction

Intracranial large artery occlusive disease (ILAO) is an important cause of ischemic stroke [1, 2], and symptomatic nonacute ILAO persisting beyond 24 h from onset is a specific form of ILAO. A considerable number of patients with this form of ILAO, especially those with hemodynamic compromise or poor collateral flow, continue to be at a higher risk for subsequent stroke despite aggressive medical therapy [3, 4]. However, the optimal treatment for medically refractory, nonacute ILAO remains unclear, with extracranial-intracranial artery bypass surgery failing to show beneficial effects in preventing ischemic attacks or stroke [5, 6]. Some recent small-sample case series have reported that endovascular recanalization is a feasible approach for carefully selected patients with symptomatic nonacute ILAO [7–15], and successful recanalization has been suggested to restore cerebral perfusion and improve the prognosis in these patients [8, 11, 16]. However, endovascular recanalization in nonacute ILAO is technically challenging and may cause potentially life-threatening complications [8–12, 14, 15, 17–21], necessitating careful selection of the patients who could benefit from this treatment. At present, patients with nonacute ILAO who show hemodynamic compromise or poor collateral flow and progressive or recurrent ischemic neurological deficits despite maximal medical therapy are considered to be the most likely to benefit from endovascular recanalization [8, 11, 13] and may be the potential candidates for endovascular recanalization. However, in these candidates, the subgroup of patients who are most likely to achieve successful recanalization remains incompletely defined, despite the development of several angiographic classification models and the identification of several predictors of technical success of recanalization for nonacute ILAO [18, 19, 21–26]. In this study, we aimed to determine the predictors of successful endovascular recanalization in patients with symptomatic nonacute ILAO, which may provide a reference for patient selection.

## Materials and methods

### Study population

We conducted a retrospective analysis of consecutive endovascular recanalization attempts in patients with symptomatic nonacute ILAO who showed hemodynamic cerebral ischemia from January 2016 to December 2022 at Shanghai Tongji Hospital affiliated to Tongji University School of Medicine. All patients signed a written informed consent prior to endovascular recanalization attempts. This retrospective review of the clinical information and radiologic records of the patients was approved by the Institutional Review Board at Shanghai Tongji Hospital, which waived the need to obtain informed patient consent for the review of the patient records and images.

Patients with symptomatic nonacute ILAO who underwent endovascular recanalization attempts were included in this study if they met the following criteria: [1] ILAO including the intracranial segments (C6, ophthalmic and C7, communicating) of the internal carotid artery (ICA), as described by Bouthiller [27], the M1 segment of the middle cerebral artery (MCA), and the intracranial vertebral artery and basilar artery diagnosed by a computed tomography angiography (CTA) or a magnetic resonance angiography (MRA) examination and confirmed by digital subtraction angiography (DSA) with thrombolysis in myocardial infarction (TIMI) grade 0 or 1 for antegrade flow through the occlusion before recanalization and estimated occlusion duration > 24 h; [2] recurrent ischemic neurological deficits (transient ischemic attack [TIA] or stroke) or progressive neurologic impairment symptoms (National Institutes of Health Stroke Scale (NIHSS) score increase ≥ 4 due to persistent or transient low perfusion despite maximal medical therapy [28]; [3] head CT or magnetic resonance imaging (MRI) showing border zone infarction and/or CT perfusion images showing a decrease in cerebral blood flow (CBF) and increase in time to peak (TTP) and mean transit time (MTT), and normal or decreased cerebral blood volume (CBV) in the territory of the culprit intracranial artery or an arterial collateral circulation grade (ACG) ≤ 3 according to the American Society of Interventional and Therapeutic Neuroradiology/ Society of Interventional Radiology (ASITN/SIR) standards [29]; and [4] brain CTA or MRA, and HR-MRI vascular wall imaging, as well as DSA confirming the presence of a vascular bed at the distal end of the occlusion, with the diameter of the occluded vessel estimated to exceed 2 mm; [5] patients with at least one risk factor for atherosclerosis (e.g., hypertension, diabetes mellitus, hyperlipidemia, coronary artery disease, cigarette smoking).

However, patients were excluded if they met any of the following criteria: [1] clinical, laboratory, or imaging findings suspicious for non-atherosclerotic occlusion, e.g., moyamoya disease or angiitis, or arterial dissection; [2] CT or angiographic evidence of severe calcification defined by an arc of calcification > 180° at the occluded segment; [3] severe angulation defined as the degree of angulation of the occluded segment > 90°; [4] known allergy or contraindication to aspirin and clopidogrel, aspirin and clopidogrel resistance, or intolerance for general anesthesia; [5] bleeding diathesis that could not be corrected; and [6] life expectancy < 2 years because of other medical conditions.

### Endovascular recanalization procedure

All procedures were performed under general anesthesia by two interventional neuroradiologists (C. F and H-Q. T) with over 10 years of experience in endovascular treatment of intracranial large artery stenosis or occlusion diseases. After placement of the sheath introducer, heparin was administered intravenously to maintain the activated clotting time between 200 and 300 s. A 6-French (6 F) guiding catheter (Navien, EV3, USA) was advanced into the cervical vertebral or ICA as high as allowed by the vessel tortuosity. If the vessels at the distal end of the occlusion could be visualized by reconstruction of the distal collateral vessel, the dual-roadmap technique was performed, in accordance with the previously described schemes for the dual-roadmap technique [20].

Under roadmap guidance, the microwire (Synchro, Stryker Neurovascular, Fremont, CA; Traxcess-14 soft-tip, MicroVention, Tustin, CA, USA) was used in combination with a microcatheter (Echelon-10, ev3 Neurovascular, Irvine, CA) to pass through the occluded segment carefully. If the microwire and microcatheter could not cross the occluded segment and enter the true distal lumen after repeated attempts, the procedure was stopped. If the microwire was successfully steered through the occluded segment, then a microcatheter injection was used to confirm the position distal to the occluded segment in the true distal lumen. Subsequently, an exchange microwire (Transend ES 014/300 Floppy, Boston Scientific Corp, Natick, MA) was placed in the appropriate anchoring position, and the microcatheter was removed. A gateway angioplasty balloon (Stryker Neurovascular, Fremont, CA) or a rapid-exchange balloon dilation catheter (Maverick, Boston Scientific, Natick, MA, USA; Neuro LPS™, Sinomed, Tianjin, China) was advanced over the exchange microwire to cross the lesion. The balloon size was based on the measurement of the proximal and distal vessels of the occlusion as well as the length of the occluded segment on the dual roadmaps. The maximum diameter of the predilation balloon was less than 80% of the estimated diameter of the lesion, and the balloon length was as short as possible but at least covering the lesion length. The balloon was slowly inflated to 6 atmospheres for 60 s. After angioplasty, based on measurement of the proximal and distal diameters of the target vessel as well as the length of the occluded segment after balloon dilation, a self-expandable stent (Enterprise, Codman & Shurtleff, Raynham, MA; LEO Baby, Balt Extrusion, Montmorency, France) or balloon-mounted stent (Apollo, MicroPort Medical, Shanghai, China) was introduced and deployed according to the operator’s preference. Postoperative angiography was performed to confirm the patency. Successful revascularization was defined as antegrade flow with a modified thrombolysis in cerebral infarction (TICI) grade ≥ 2b and residual stenosis of ≤ 50% [22]. Brain CT was performed immediately after the operation to rule out intracranial hemorrhage, after which all patients were typically monitored in neuro critical care units for 24 h postprocedure with a target systolic blood pressure < 120 mm Hg to reduce the risk of reperfusion hemorrhage [10, 18–20, 24].

Dual antiplatelet therapy with clopidogrel (75 mg) and aspirin (100 mg) was initiated at least 3 days prior to the recanalization. The patients who received clopidogrel (75 mg) and aspirin (100 mg) for < 3 days were given 300-mg loading doses of clopidogrel and aspirin prior to the recanalization. Platelet reactivity was evaluated by thromboelastography. Aspirin resistance was defined as < 50% inhibition of arachidonic acid–induced platelet aggregation, and clopidogrel resistance was considered as < 30% inhibition of ADP–induced platelet aggregation [30]. Patients who showed clopidogrel resistance were treated with ticagrelor 90 mg twice a day. Dual antiplatelet therapy was maintained for 3–6 months, with life- long aspirin or clopidogrel monotherapy maintained thereafter. After the procedure, risk factor control was based on the AHA/ASA guidelines and the SAMMPRIS trial protocol [31, 32], and rehabilitation training was prescribed if necessary. After discharge, clinical follow-ups of all patients were conducted by telephone or clinic visit at 30 days. Subsequently, clinical and angiographic follow-up assessments were recommended, the follow-up strategies were similar to those described in our previous study [8].

### Data collection and definition of potential variables associated with successful recanalization

Cases were identified through a search of the prospectively acquired endovascular databases at our institution. Detailed data on demographic and baseline clinical characteristics, preoperative radiological features, including occlusion site, stump morphology, occlusion length, calcification and angulation at the occluded segment, slow distal antegrade flow (SDAF) sign, and bridging collateral vessels, procedural results (modality of recanalization, technical success, postprocedural perfusion status), and periprocedural complications, as well as follow-up outcomes (clinical and angiographic outcomes) were collected. All image assessments were conducted by two independent neuroradiologists, and any discrepancies were resolved by consensus. The duration of occlusion was estimated on the basis of clinical events such as the sudden onset or worsening of ischemic symptoms or was determined on the basis of the patients’ previous imaging examination results, and was categorized as ≤ 3 months or > 3 months. The time from imaging-documented occlusion to endovascular intervention was recorded. The occlusion site was categorized as intracranial ICA, M1 segment of the MCA, basilar artery, or intracranial vertebral artery. The presence or absence of an occlusion stump and stump morphology were evaluated on DSA. A stump was considered to be present if contrast filling was observed within the residual vessel proximal to the occluded segment [25]. Angiographic morphology of the stump was classified as “tapered” if the occluded segment ended in a funnel-shaped form or “blunt” if it did not, consistent with the definitions used in percutaneous coronary intervention (PCI) for chronic total occlusion (CTO) lesions [33]. The length and angle of the occluded segment were measured on multiple planar reconstruction images obtained from the preoperative CTA original images using the Aquilion ONE (Toshiba medical systems, Tokyo, Japan) postprocessing work station. The occluded segment was the segment that was completely invisible and showed a total luminal filling defect on multiple planar reconstruction (MPR) images in CTA. The occlusion length was automatically calculated by the software after we manually traced the longitudinal axis of the occluded segment, which was recorded and categorized as < 10 mm or ≥ 10 mm. The occlusion angle was defined as the angle between the proximal longitudinal axis and the distal longitudinal axis at the occluded segment, with a straight occluded segment defined as 0°, which was measured after we manually traced the proximal and distal longitudinal axes of the occluded segment, and was categorized as > 45° or ≤ 45°. The calcification at the occluded segment was evaluated on preoperative CTA original images and MPR images and categorized as no evident calcification or the presence of calcification. The SDAF sign was defined as slow antegrade contrast opacification distal to the occlusion site on the delayed images of the presenting arteriogram, as previously described by Christoforidis et al. [34], and categorized as no SDAF sign or presence of SDAF sign. The presence of bridging collateral vessels was angiographically defined as a plexus of micro-vessel channels bridging between the vessel proximal and distal to the occlusion, which allowed antegrade opacification of the patent vessels distal to the occlusion [35, 36].

### Statistical analysis

The continuous normally distributed quantitative variables were expressed as mean ± standard deviation (SD); non-normally distributed variables were expressed as the median and interquartile range; and categorical variables were expressed as number and percentage. Differences in categorical variables between the successful recanalization group and the failure group were assessed with the χ^2^ test or Fisher’s exact test. Differences in continuous variables between the two groups were assessed using the t test or Mann–Whitney U test.

The association between clinical and radiological characteristics and the technical success was assessed with univariate logistic regression analysis. All variables with a p-value < 0.10 in the univariate analysis were entered into the multivariate logistic regression model. A backward stepwise selection was performed to select independent predictors using a likelihood ratio test with Akaike information criterion (AIC) as the stopping rule. A p-value < 0.05 was required for all variables to be included in the final multivariate stepwise model. Two-sided P values ≤ 0.05 were considered statistically significant. All statistical analyses were performed using commercial SPSS 20.0.

## Results

### Patients’ baseline clinical characteristics and radiological features of the lesions

Seventy patients with symptomatic nonacute ILAO who underwent endovascular recanalization attempts were included in this study. The mean ± SD age was 60 ± 9.6 years, and the study population included 48 (68.6%) male and 22 (31.4%) female patients. Demographic and baseline clinical characteristics of these patients are listed in Table 1. The lesion characteristics at the occluded segment on preoperative radiological assessments are summarized in Table 2. Among the 70 patients, 28 (40%) had progressive stroke, 23 (32.9%) had recurrent ischemic stroke, and 19 (27.1%) had recurrent TIAs. The occluded sites were within the intracranial ICA in eight (11.4%) patients, the M1 segment of the MCA in 46 (65.7%) patients, the basilar arteries in six (8.6%) patients, and the intracranial vertebral artery in 10 (14.3%) patients. Median duration of occlusion was 12.5 d (interquartile range [IQR], 3–34 d). The median interval between imaging-documented occlusion and intervention was 8.5 d (IQR, 3–28 d). A total of 50 (71.4%) patients showed a tapered stump at the proximal end of the occlusion. The median occlusion length was 8.4 mm (7.8–9.25 mm), while the occlusion length was < 5 mm in 11 patients, 5–10 mm in 44 patients, and > 10 mm in 15 patients. The median (IQR) occlusion angle was 15° (7.5°-23.25°), while the occlusion angle was ≥ 45° in 3 (4.3%) patients. Calcification of the occlusion was observed in four (5.7%) patients. Forty-one (58.6%) patients showed an SDAF sign, while four (5.7%) patients showed bridging collateral vessels.

**Table 1:** Baseline demographic and clinical characteristics of the patients

Variable	Total (n = 70)	Successful group (n = 57)	Failed group (n = 13)	p Value
Age, mean (SD), yr	60 ± 9.6	59.7 ± 9.4	60.1 ± 9.7	0.9034
Male, n (%)	48(68.6)	42(73.7)	6(46.2)	0.1099
Risk factors, n (%)				
Hypertension	54(77.1)	45 (78.9)	9 (69.2)	0.6988
Diabetes mellitus	30(42.9)	26(45.6)	4(30.8)	0.3291
Coronary heart disease	9(12.9)	7(12.3)	2(15.4)	0.8749
Smoking history	39(55.7)	34(59.6)	5(38.5)	0.1652
Alcohol history	20(35.1)	16(28.1)	4(30.8)	0.8841
Dyslipidemia	33(57.9)	29(50.9)	4(30.8)	0.1900
Qualifying event, n (%)				
Progressive stroke	28(40)	25(43.9)	3(23.1)	0.1675
Recurrent stroke/TIA	42(60)	32(56.1)	10(76.9)	
Estimated occlusion duration (d), median (IQR)	12.5(3, 34)	9(3, 30)	60(13.5, 93.5)	0.0003
Estimated occlusion duration, n (%)				
≤ 3 months	65(92.9)	56(98.2)	9(69.2)	0.0035
> 3 months	5(7.1)	1(1.8)	4(30.8)	
Time from imaging-documented occlusion to intervention(d), median (IQR),	8.5(3,28)	5(2,24.5)	52(9.5,88.5)	0.0007

IQR, interquartile range; SD, standard deviation, TIA, transient ischemic attack

**Table 2:** Radiological characteristics of the lesions

Variable	Total (n = 70)	Successful group (n = 57)	Failed group (n = 13)	p Value
Responsible artery				
Anterior circulation	54(77.1%)	44(77.2%)	10(76.9%)	0.7300
Posterior circulation	16(22.9%)	13(22.8%)	3(23.1%)	
Stump condition				
Tapered stump	50(71.4%)	46(80.7%)	4(30.8%)	0.0011
No stump or blunt	20(28.6%)	11(19.3%)	9(69.2%)	
Occlusion length (mm) median (IQR),	8.4(7.8,9.25)	8.2(7.2,8.8)	12.8(8.5,15.5)	< 0.0001
Occlusion length				
< 10 mm	56(80%)	50(87.7%)	6(46.2%)	0.0027
≥ 10 mm	14(20%)	7(12.3%)	7(53.8%)	
Occlusion angle°, median (IQR),	15(7.5,23.25)	14.5(7.5,22.25)	15(8.75,27.5)	0.4068
Occlusion angle°				
≥ 45°	3(4.3%)	2(3.5%)	1(7.7%)	0.4655
< 45°	67(95.7%)	55(96.5%)	12(92.3%)	
Occlusion calcification				
Absent	66(94.3%)	55(96.5%)	11(84.6%)	0.1543
Present	4(5.7%)	2(3.5%)	2(15.4%)	
Slow antegrade flow distal to the occlusion site				
Absent	29(41.4%)	18(31.6%)	11(84.6%)	0.0005
Present	41(58.6%)	39(68.4%)	2(15.4%)	
Bridging collateral vessels				
Absent	66(94.3%)	56(98.2%)	10(76.9%)	0.0186
Present	4(5.7%)	1(1.8%)	3(23.1%)	

IQR, interquartile range; SD, standard deviation;

### Primary procedure results and periprocedural complications

Technically successful recanalization was achieved in 57 patients (81.4%), of which 15 showed TICI grade 2b reperfusion and 42 showed TICI grade 3 reperfusion. Among these patients, three underwent balloon angioplasty alone. Balloon-mounted stents were used in nine patients and self-expanding stents were used in 45 patients after balloon angioplasty. Endovascular recanalization failed in 13 patients, of which 11 showed TICI grade 0 reperfusion and two showed TICI grade 2a reperfusion. Among the 13 patients showing recanalization failure, the microwire could not pass through the occluded segment and enter the true lumen in 10 patients; one of these patients experienced vascular dissection and perforation during the recanalization attempts, which caused subarachnoid hemorrhage (SAH) and necessitated coil embolization of the perforation. Two patients achieved TICI grade 2a antegrade flow due to residual stenosis of > 50% immediately after stent implantation, although the microwire could successfully traverse the occluded segments. One patient experienced intracranial hemorrhage (ICH) of the distal lenticulostriate artery after the microwire successfully traversed the occluded segments and pre-dilation with balloon was performed during the procedure, necessitating coil embolization of the recanalized occlusion portion.

The rate of periprocedural complication was 21.4% (15 of 70), the overall 30-day morbidity and mortality rates were 7.1% (5 of 70) and 2.9% (2 of 70), respectively. As shown in Table 3, the periprocedural complications include two cases of asymptomatic dissection, four cases of ICH and SAH, four cases of symptomatic distal embolism, two cases of hyperperfusion syndrome and one case of perforator occlusion. In the group of successful recanalization, one patient experienced ICH and SAH related to vascular dissection or laceration following mechanical thrombectomy; the patient underwent craniotomy evacuation of hematoma and eventually survived with neurologic dysfunction. Four patients suffered from symptomatic distal embolism that completely disappeared without neurological sequelae after aggressive medical therapy. Two patients experienced ICH as a result of hyperperfusion syndrome within 24 h. The two patients died one week after craniotomy evacuation of hematoma and decompressive craniectomy. One patient showed perforator occlusion of the basilar artery that was complicated with locked-in syndrome. In the failed group, 2 cases experienced asymptomatic dissection due to the inability of the microwire to traverse the occluded segment. Three patients experienced ICH and SAH. Among the 3 patients, two patients experienced SAH related to vascular dissection and perforation due to microwire manipulation during the recanalization attempts and did not show neurological sequelae after coil embolization of the dissection and temporary occlusion with a balloon.Another one experienced ICH due to rupture of the distal lenticulostriate artery after the microwire had successfully traversed the occluded segments and pre-dilation with balloon was performed; coil embolization of the recanalized occlusion portion was performed, and the patient survived with moderate neurologic deficits after aggressive medical therapy. Two patients who achieved TICI grade 2a antegrade flow developed in-stent thrombosis and re-occlusion after the procedur*e*, and the two patients experienced symptom fluctuation and worsening.

**Table 3:** Periprocedural complications based on the technical result and occlusion site of endovascular recanalization

Complications	Patients (n = 70)	Recanalization group		Recanalization site	
		Successful group (n = 57)	Failed group (n = 13)	Anterior circulation (n = 54)	Posterior circulation(n = 16)
Dissection	2(2.9%)	0(0%)	2(15.4%)	0(0%)	2(12.5%)
ICH and/ or SAH	4(5.7%)	1(1.8%)	3(23%)	4(7.4%)	0(0%)
Distal embolism	4(5.7%)	4(7%)	0(0%)	4(7.4)	0(0%)
In-stent thrombosis	2(2.9%)	0(0%)	2(15.4%)	2(3.7%)	0(0%)
Hyperperfusion syndrome	2(2.9%)	2(3.5%)	0(0%)	1(1.9%)	1(6.3%)
Perforator occlusion	1(1.4%)	1(0%)	0(0%)	0(0%)	1(6.3%)
Morbidity within 30 days	5(7.1%)	2(5.3%)	3(23.1%)	4(7.4%)	1(6.3)
Mortality within 30 days	2(2.9%)	2(3.5%)	0(0%)	1(1.9%)	1(6.3%)

ICH, intracerebral hemorrhage;SAH, Subarachnoid hemorrhage

### Clinical and angiographic follow-up outcomes

The 3-month clinical follow-up data were available for 66 patients. Follow-up data were available for 55 of the 57 patients who underwent successful recanalization, and these patients showed no recurrence of TIA or stroke, except in one patient who developed in-stent stenosis and showed TIA. At the 3-month follow-up, the mRS score was favorable (mRS score = 0–2) in 51/55 (92.8%) patients, moderate (mRS score = 3) in two patients, and poor (mRS score = 4–5) in two patients. Neurological status deteriorated in two patients, of which one experienced ICH due to vascular dissection or laceration following mechanical thrombectomy and the other experienced locked-in syndrome due to perforator occlusion of the basilar artery. The neurological status was stable in 16 patients and improved in 37 patients. Clinical follow-up data were available for 11 of the 13 patients who showed recanalization failure; among these patients, one showed recurrent ischemic stroke and three experienced TIA. None of these patients consented to further extracranial-intracranial artery bypass surgery. Three patients experienced symptom deterioration; among them, two patients with a TICI 2a grade antegrade flow after the procedure developed in-stent thrombosis and re-occlusion, while the third patient showed ICH due to rupture of the distal lenticulostriate artery. Four patients presented with a stable neurological status. At the 3-month follow-up period, the mRS score was favorable in six patients (54.5%), moderate in two patients, and poor in three patients with symptom deterioration. The proportion of favorable function outcome was higher in the successful group than in the failed group (92.8% vs. 54.5%, P = 0.0039); in contrast, the rate of the neurological deteriorated was lower in the successful group than in the failed group (3.6% vs. 27.3%, P = 0.0375).

Angiographic follow-up data at 3 months were available in 55 patients, including 53 of the 57 patients who underwent successful recanalization. At the 3-month angiography follow- up, one patient who experienced TIA developed in-stent restenosis, and two patients developed asymptomatic in-stent re-occlusion. Of the 13 patients showing recanalization failure, angiographic follow-up data were available for two patients with TICI grade 2a antegrade flow after the procedure, and both showed re-occlusion, while the remaining 11 patients refused to undergo further radiological examination.

### Comparison of baseline clinical and radiological characteristics between the groups showing successful recanalization and recanalization failure

**Fig. 1:**
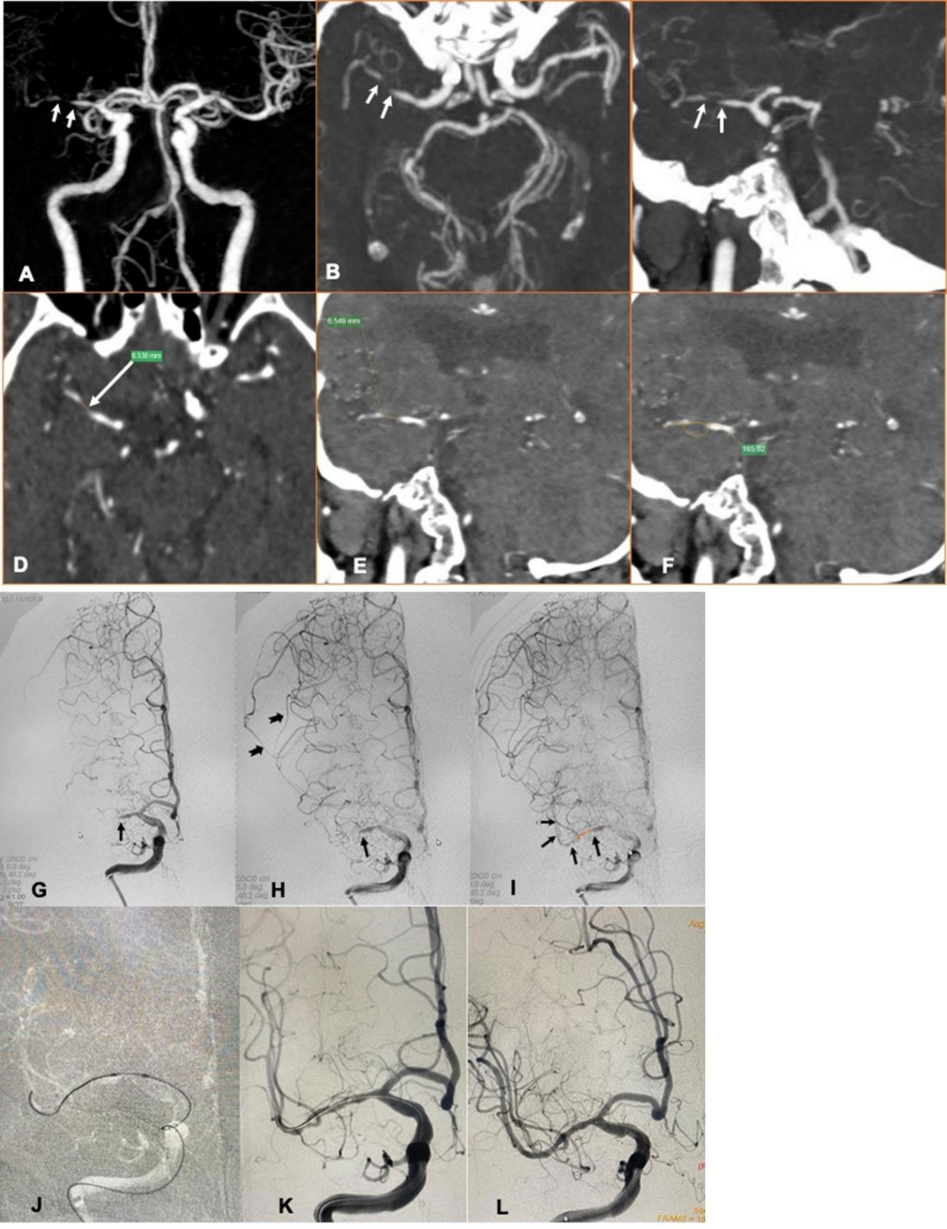
A patient with symptomatic middle cerebral artery (MCA) occlusion underwent endovascular recanalization that led to successful recanalization. **A-C**: Preprocedural computed tomography angiography (CTA) volume rendering (VR) reconstruction (**A**) and maximal intensity projection (MIP; **B** and **C**) images showed an interrupted continuity in the M1 segment of the right MCA (white arrow). **D** and **E**: Multiple planar reconstruction (MPR) of CTA images showed the total luminal filling defect at the occluded segment of the MCA; the occlusion length was 6.5 mm. **F**: MPR of CTA images showed the occluded segment made a 15° turn from the proximal to the distal M1 segment. **G-I**: Digital subtraction angiography (DSA) confirmed occlusion of M1 segment of the right MCA (**G**), a tapered stump of the occluded segment (black arrow), and a slow antegrade flow through the occluded segment (TIMI grade 1) and visualization of the vascular bed at the distal end of the occlusion (black dovetail arrow), as well as retrograde blood flow (black dovetail arrowhead) from the anterior cerebral artery on the midarterial-phase (**H**) and late arterial-phase images (**I**). **J**: After successful navigation of the microwire and microcatheter through the occlusion, a 1.5 × 10 mm rapid-exchange balloon (Neuro LPS™, Sinomed, Tianjin, China) was positioned to the occluded segment (**K**), after which the lesion was predilated with 1.5 × 10 mm and 2.25 × 10 mm balloons and a 2.5 × 18 mm LEO baby stent was implanted. **L**: Postprocedural DSA demonstrated technically successful recanalization with TICI grade 3

**Fig. 2:**
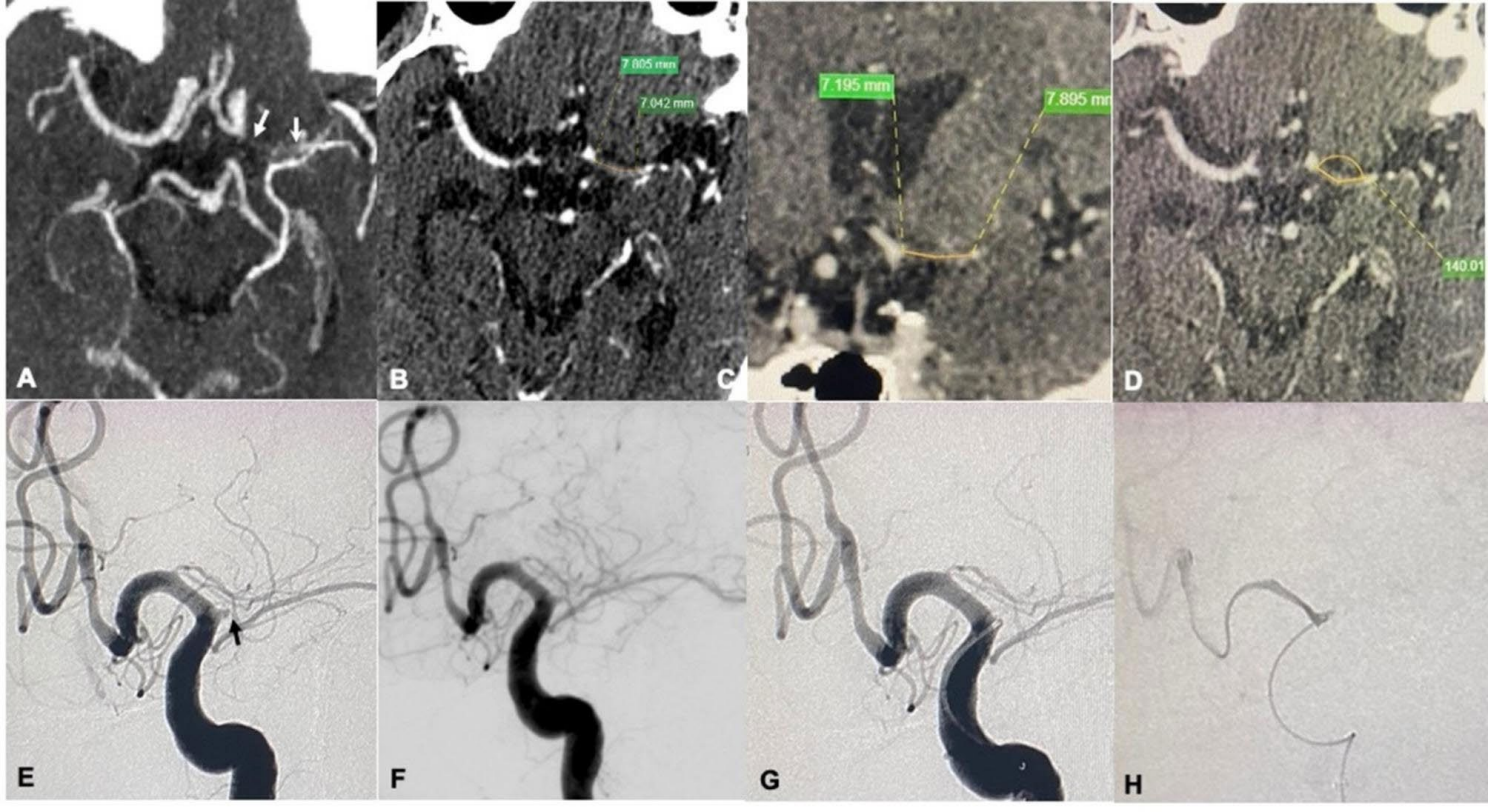
A patient with symptomatic middle cerebral artery occlusion (MCA) underwent endovascular recanalization attempts that were unsuccessful because the guidewire could not be advanced through the occlusion. **A**: Preprocedural computed tomography angiography (CTA) maximal intensity projection (MIP) images showed an occlusion at the origin of the left MCA and the proximal site (white arrow) and the distal site (white dovetail arrow) of the occluded segment. **B** and **C**: Multiple planar reconstruction (MPR) images of CTA images obtained from the axial (**B**) and oblique (**C**) views showed the total luminal filling defect at the occluded segment of the MCA. The occlusion length was approximately 15 mm. **D**: The occluded segment made a 40° turn from the proximal to the distal M1 segment. **E** and **F**: Digital subtraction angiography (DSA) confirmed occlusion of the left MCA origin (**E**), a short blunt stump of the occluded segment (white arrow), and no slow antegrade flow through the occluded segment on the mid-late arterial phase (**F**). **G**: The microwire and microcatheter were positioned to the origin of the left MCA, and attempts were made to pass through the occluded segment. **H**: After repeated attempts, the microwire and microcatheter could not cross the occluded segment, the injection by microcatheter showed no dissection and extravasation of contrast media, and the procedure was stopped

The successful recanalization group and recanalization failure groups showed no significant differences in age, sex, vascular risk factors, or distributions of the qualifying events and the responsible arteries. The successful recanalization group showed a shorter median occlusion duration (9 vs. 60 d, p = 0.0003) and a shorter interval between imaging-documented occlusion and intervention (5 vs. 52 d, p = 0.0007). The proportion of patients with occlusion duration ≤ 3 months was greater in the successful recanalization group than in the failure group (98.2% vs. 69.2%, p = 0.0035). The median occlusion length was shorter in the successful recanalization group than in the failure group (8.2 vs. 12.8 mm, p < 0.0001). The successful recanalization group also included a larger proportion of patients with occlusion length < 10 mm (87.7% vs. 46.2%, p = 0.0027). The occlusion angle and the incidence of occlusion calcification showed no significant difference between the two groups. The successful recanalization group included a greater proportion of patients with a tapered stump (80.7% vs. 30.8%, p = 0.0011) and slow antegrade flow distal to the occlusion site (68.4% vs. 15.4%, p = 0.0005) and a smaller proportion of patients with bridging collateral vessels (1.8% vs. 23.1%, p = 0.0186). Typical cases of successful recanalization and recanalization failure are shown in Figs. 1 and 2, respectively.

### Univariate and multivariate analysis of the predictors of technical success

Univariate analysis was performed with logistic regression to determine associations with technical success (Table 4). The technical success rate was greater in patients with occlusion duration ≤ 3 months (odds ratio [OR]: 24.889; 95% confidence interval [CI]: 2.491-248.638), a tapered stump (OR: 9.409; 95% CI: 2.442–36.460), occlusion length < 10 mm (OR: 8.333; 95% CI: 2.167–32.052), and slow antegrade flow distal to the occlusion site (OR: 11.917; 95% CI: 2.390-59.427). However, the technical success rate was lower in patients with bridging collateral vessels (OR: 0.060; 95% CI: 0.006–0.631).

**Table 4:** Logistic regression analysis of the predictors of technical success

Variable	0dds ratio	95% Confidence interval	P Value
Hypertension	0.600	0.157–2.289	0.455
Diabetes mellitus	0.530	0.146–1.921	0.334
Coronary heart disease	0.770	0.140–4.222	0.763
Smoking history	0.423	0.123–1.456	0.172
Alcohol history	0.878	0.236–3.260	0.846
Dyslipidemia	0.429	0.118–1.555	0.198
Qualifying event (Progressive stroke)	2.604	0.647–10,479	0.178
Estimated occlusion duration (≤ 3 months)	24.889	2.491-248.638	0.006
Occlusion site (anterior circulation)	1.015	0.243–4.237	0.983
Tapered stump	9.409	2.442–36.460	0.001
Occlusion length (< 10 mm)	8.333	2.167–32.052	0.002
Occlusion angle°>45°	0.436	0.037–5.213	0.512
Occlusion calcification (presence)	0.200	0.025–1.576	0.126
Slow antegrade flow distal to the occlusion site	11.917	2.390-59.427	0.003
Bridging collateral vessels (presence)	0.060	0.006–0.631	0.019

Multivariate logistic analysis using stepwise backward logistic regression was performed to determine independent predictors for technically successful recanalization. After adjusting for age and sex, occlusion duration ≤ 3 months (OR: 22.529; 95% CI: 1.636-310.141; P = 0.020), the presence of a tapered stump (OR: 7.498; 95% CI: 1.533 to 36.671; P = 0.013), and occlusion length < 10 mm (OR: 7.049; 95% CI: 1.402 to 35.441; P = 0.018) were identified as independent positive predictors of technical success of endovascular recanalization for nonacute ILAO (Table 5).

**Table 5:** Multivariate analysis of the predictors of technical success Using Multiple Backward Stepwise Selection with Age and Sex Adjusted

Variable	Adjust odds ratio	95% Confidence interval	P Value
Estimated occlusion duration (≤ 3 months)	22.529	1.636-310.141	0.020
Tapered stump	7.498	1.533–36.671	0.013
Occlusion length (< 10 mm)	7.049	1.402–35.441	0.018

## Discussion

In this study, we found that successful recanalization was associated with occlusion duration, stump morphology, occlusion length, SDAF sign, and the presence of bridging collaterals. The technical success rate was higher in patients with occlusion duration ≤ 3 months, a tapered stump, occlusion length < 10 mm, and SDAF sign. However, the technical success rate was lower in patients with bridging collaterals. Multivariate analysis further showed that occlusion duration ≤ 3 months, a tapered stump, and occlusion length < 10 mm were independent positive predictors for technical success in endovascular recanalization for nonacute ILAO. These findings may help predict the likelihood of successful recanalization in patients with symptomatic nonacute ILAO and also provide a reference for the selection of appropriate patients. Patients with positive predictors for technical success appear to be the best candidates for endovascular recanalization. To the best of our knowledge, this is the first case series to determine the predictors of successful recanalization in patients with symptomatic nonacute ILAO. In addition, our results suggested that successful recanalization might effectively improve the degree of disability of symptomatic non-acute ILAO patients. A systematic pre-procedural evaluation is therefore important to identify patient and lesion characteristics that carry higher success rates.

In our study, 57 (81.4%) patients achieved technical success in endovascular recanalization; the periprocedural complication rate was 21.4% (15 of 70), and the overall 30-day morbidity and mortality rates were 7.1% (5 of 70) and 2.9% (2 of 70), respectively. Our technical success and periprocedural complication rates were comparable to those in recently reported case series [7, 8, 12, 13, 18–23, 26]. The technical success and periprocedural complication rates for endovascular recanalization of nonacute ILAO have been reported to range from 53.1 to 100% and 4-44.4%, respectively [9–13, 15, 17–21, 23, 24], while the 30-day stroke and mortality rate has been reported to be 3.8-16.7% [11, 18–21, 23]. The differences in the successful recanalization and periprocedural complication rates in these studies may be due to differences in operator experience, intracranial occlusion location, occluded segment characteristics, the interval from vessel occlusion to endovascualr recanalization, and the roadmap techniques used. In this study, the major reason for recanalization failure was that the microwire could not traverse the occluded segment. The two operators in this study had over 10 years of experience in endovascular treatment for intracranial large artery stenosis or occlusion. If the vessels at the distal end of the occlusion could be visualized by reconstruction of the distal collateral vessel, the dual-roadmap technique was performed. Therefore, operator experience and the use of the roadmap technique had little influence on recanalization failure, and the duration of occlusion and the features of the occluded segment may be major factors influencing the success of recanalization.

The occlusion duration is usually considered a key factor determining the success of recanalization. The occlusion duration may influence the histological components and the occlusion length. The longer the occlusion duration, the greater the amount of fibrous tissue and the degree of calcification in the lesion, which may cause hardening of the lesion; moreover, longer occluded segments will also show a progressive thrombotic process, reducing the likelihood of recanalization. A large number of studies have demonstrated that occlusion duration < 3 months is an independent predictor for initial successful recanalization in PCI for CTO [35, 37]. However, few studies have investigated the effects of occlusion duration on the technical success of endovascualr recanalization in patients with symptomatic nonacute ILAO. Several recent studies on endovascualr recanalization for nonacute ILAO showed that patients with successful recanalization had a shorter occlusion time, suggesting that the occlusion duration may be associated with technical success [11, 24, 26], but this association was not found in other studies [22]. These inconsistencies may be partly attributable to the difficulty and inherent limitations of estimating the duration of occlusion from clinical information. In this study, the occlusion duration was estimated on the basis of the definition of the estimated duration for CTO [35, 37, 38]. The occlusion duration represented the approximate time of artery occlusion. Therefore, our results reflect the effect of occlusion duration on technical success. In the present study, occlusion duration ≤ 3 months was identified as an independent positive predictor for technical success, which was consistent with the findings of a recent study [24]. However, our results may be arbitrary because the exact cut-off value of the occlusion duration for predicting technical success and the optimal timing of endovascualr recanalization for nonacute ILAO remains unclear. Further investigation of this factor is warranted.

The presence or absence of a stump and stump morphology are two important determinants of endovascualr recanalization. Experiences with the carotid and coronary artery in the treatment of CTO have indicated that the presence of a stump and a tapered stump at the occlusion site are favorable for successful recanalization [33, 35, 37–42]. Occlusions with a tapered stump frequently show histologic features favorable for PCI, such as small-lumen recanalized areas, surrounding loose fibrous tissue, and a short occluded segment [43]. These features may help the guide wire easily enter the occluded segment. In contrast, in occlusions without a vessel stump, the vessel lumen is completely occluded from the origin of the vessel, and in the absence of a target point for exploration and a landing zone for the microwire, the microwire would easily enter the branch vessel proximal to occlusion. Similarly, in occlusions with blunt stump, the vessel stump proximal to the occlusion is blunt, and small-lumen recanalization and short occluded segments are rarely observed [43]. As a result, the tip of the guidewire could easily turn back and loop and be unable to enter the occluded segment. At present, the influence of stump morphology on recanalization in patients with ILAO remains unclear. Chao et al. [24] investigated factors related to technical success, and found that a tapered stump was an independent predictor for successful recanalization of non-acute occluded ICA. However, in another study, no significant difference was observed between the recanalization success rates for tapered and non-tampered stumps, which was attributed to partial neutralization by occlusion duration (< 3 months) [18]. In this study, the stump morphology was closely related to the success rate of recanalization, and a tapered stump was an independent positive predictor for technical success, which is consistent with the results of most previous studies on this topic [24, 25]. However, a study with a large sample size is needed to validate this finding.

Occlusion length, occlusion angle, and occlusion calcification are known to affect the success rate in PCI for CTO [33, 35, 37–39, 41, 42]. The longer the occlusion length, the larger the angulation of the occluded segment and the more severe the occlusion calcification, and the lower the success rate of endovascular recanalization. Accumulated evidence from a large number of studies on PCI for CTO have revealed that occlusion length > 15 mm, occlusion angle > 45°, and moderate-to-severe occlusion calcification were independent predictors of technical failure in endovascular recanalization for this disease [33, 35, 39–41]. In this study, occlusion length < 10 mm was identified as an independent positive predictor for technical success of recanalization, which is equivalent to the length of type I occlusion according to the new angiographic classification proposed by Gao et al. [18, 19, 21]. However, in this study, we did not observe a significant association of occlusion angulation and occlusion calcification with the technical success of recanalization. One possible reason for this finding may be that patients with the severe angulation and calcification of the occluded segment were excluded and the majority of patients with larger occlusion angles and moderate-to-severe occlusion calcification were infrequently referred for endovascular treatment, which might have resulted in a statistically negative result. Therefore, a larger number of cases would be required to clarify whether an inverse relationship does exist between the angulation and calcification of the occluded segment and the success rate of recanalization. In our study, the occlusion length, occlusion angle, and occlusion calcification were routinely evaluated in advance by MPR images of CTA. HR-MRI was also performed to evaluate the characteristics of the occluded segments, such as occlusion morphology, angle, and length. In a previous study, the occlusion length judged by cerebrovascular angiography was measured mainly on the basis of the distance between proximal occlusion and reconstruction of distal collateral vessels and was measured as a straight line rather than the actual distance between the proximal and distal sites of the occluded segments on the angiogram [18, 21, 23, 25]; thus, the measured occlusion length may be longer than the true length of underlying atherosclerotic lesions [15]. In our study, the occlusion length was automatically calculated by the software after manually tracing the longitudinal axis of the occluded segment on MPR images of CTA; therefore, the occlusion length measurements in our study may be much closer to the actual occlusion length than that measured on DSA. Similarly, the occlusion angle measurements obtained in our study may more accurately reflect vessel course of the occluded segment, and the degree of occlusion calcification we assessed may be more precise.

The presence of bridging collateral vessels in CTO is a strong predictor of procedural failure [35, 37, 40, 42]. Bridging collateral vessels are well-developed vaso vasorum that appear as a dense plexus of micro-vessels typically extending from the adventitia through the media into the thickened intima, and their development appears to be proportional to the duration of occlusion [35]. The vessel wall of bridging collateral vessels surrounding chronically occluded lesions is fragile and prone to perforation during angioplasty [44]. Therefore, coronary angioplasty of such lesions has been assumed to result in an unfavorable outcome. Our study is, to our knowledge, the first to investigate the effects of bridging collateral vessels on recanalization for nonacute ILAO. Although our results revealed that the presence of bridging collateral vessels was associated with procedural failure, multivariate analysis did not identify it as an independent predictor of procedural failure. This may be attributable to the fact that our study included only a few patients with such vessels because patients with such unfavorable anatomic features were infrequently referred for endovascular treatment. However, it should be emphasized that the presence of bridging collateral vessels shoud still be evaluated before procedure. Future studies with a large sample size are required to validate this result.

The appearance of the SDAF sign on advanced-stage cerebrovascular arteriograms obtained before intra-arterial thrombolytic treatment (IAT) is known to be a statistically significant predictor for recanalization [34]. IAT can achieve more favorable recanalization in patients with the SDAF sign than in patients without it. The SDAF sign may be the result of microchannel formation within thrombi after occlusion or extremely high-grade stenosis of the arterial lumen (pseudo-occlusion) [45]. The presence of the SDAF sign may delineate roughly morphological features of occlusion, such as the course of occlusion and occlusion length. Therefore, the appearance of the SDAF sign on advanced-stage cerebrovascular arteriograms can guide the navigation of the microwire along the course of occlusion and indicate whether the tip of the microwire has deviated from the direction of the vascular longitudinal axis, thereby decreasing the risk of vascular perforation and dissection. In addition, it can indicate whether the tip of the microwire is traversing the occluded segment and landing on the normal vessel bed distal to occlusion. To the best of our knowledge, this study is the first to investigate the effects of the SDAF sign on recanalization in patients with nonacute ILAO. Our results showed that the SDAF sign was closely associated with the technical success rate of recanalization; however, in multivariate analysis, it was not identified as an independent predictor of successful recanalization. One possible cause for this discrepancy may be that the influence of occlusion duration and stump morphology on successful recanalization undermined the association of the SDAF sign to occlusion with successful recanalization. Future studies with larger case numbers would be necessary to validate whether the SDAF sign can independently predict technical success of recanalization for symptomatic nonacute ILAO.

### Limitations

This study had several limitations. First, this was a single-center retrospective study with a relatively small sample size,and the potential selection bias may have obscured some relevant factors. The regressions’ outputs display the OR with very broad confidence intervals, even if there is statistical significance, the strength of the association between predictors and successful recanalization may be overestimated. Therefore, the findings of this study should be interpreted with caution. A prospective study with a large sample size is required to validate our findings. Second, the occlusion sites in this study included the intracranial ICA, M1 segment of the MCA, intracranial vertebral segment, as well as the basilar artery. Although the technical difficulty and risk of endovascular recanalization may vary among different sites of ILAO [7, 11, 25], we did not compare the findings for different intracranial arteries because of the small sample size. A subgroup analysis in a large-cohort study should be performed to determine whether ILAOs at different sites have different predictors of recanalization. Third, in accordance with previous studies, we set 3 months as the cut-off value for occlusion duration and 10 mm as the cut-off value for occlusion length in the present study to determine their predictive power for recanalization [18, 19, 21]; however, these values may be slightly arbitrary because the findings could not clarify whether the cut-off values of the occlusion duration and occlusion length differ across different occlusion sites. A recent series of studies showed that the success rate of recanalization was significantly higher in patients with an occlusion length < 10 mm who showed retrograde collateral filling to the distal vascular bed when the occlusion site was located at the intracranial ICA, M1 segment of the MCA, and the basilar artery [18, 19, 21]. In contrast, when the intracranial vertebral artery was occluded, occlusion length > 15 mm was one of the factors leading to recanalization failure [23]. Therefore, the cut-off values of occlusion duration and occlusion length for predicting successful recanalization require further investigation. Fourth, while factors such as bridging collateral vessels and the SDAF sign, which were previously identified as independent predictors of recanalization, were found to be associated with technical success of recanalization in our study, they are not identified as independent predictors for recanalization. A large-scale, multiple-center clinical study is required to further validate whether these factors can independently predict the technical success of recanalization.

## Conclusions

Occlusion duration ≤ 3 months, tapered stump, and occlusion length < 10 mm were identified as independent positive predictors of technical success in endovascular recanalization for symptomatic nonacute ILAO. These findings may help predict the likelihood of successful recanalization in patients with symptomatic nonacute ILAO and also provide a reference for the selection of appropriate patients. Patients with positive predictors for technical success appear to be the best candidates for endovascular recanalization, but further prospective and multicenter studies are required to validate our findings.

## Data Availability

The datasets used and/or analysed during the current study are available from the corresponding author on reasonable request.

## Abbreviations

ILAO: Intracranial large artery occlusion
OR: Odds ratio
CI: Confidence interval
ICA: Internal carotid artery
MCA: Middle cerebral artery
CTA: Computed tomography angiography
MRA: Magnetic resonance angiography
DSA: Digital subtraction angiography
TIMI: Thrombolysis in myocardial infarction
TICI: Thrombolysis in cerebral infarction
TIA: Transient ischemic attack
NIHSS: National Institutes of Health Stroke Scale
mRS: Modified Rank score
MRI: Magnetic resonance imaging
CBF: Cerebral blood flow
TTP: Time to peak
MTT: Mean transit time
CBV: Cerebral blood volume
ACG: Arterial collateral circulation grade
ASITN/SIR: American Society of Interventional and Therapeutic Neuroradiology/ Society of Interventional Radiology
SDAF: Slow distal antegrade flow
PCI: Percutaneous coronary intervention
CTO: Chronic total occlusion
MPR: Multiple planar reconstruction
SD: Standard deviation
IQR: Interquartile range
SAH: Subarachnoid hemorrhage
ICH: Intracranial hemorrhage
IAT: Intra-arterial thrombolytic treatment

## References

[1] Gorelick PB, Wong KS, Bae HJ, Pandey DK (2008). Large artery intracranial occlusive disease: a large worldwide burden but a relatively neglected frontier. Stroke.

[2] Smith WS, Lev MH, English JD, Camargo EC, Chou M, Johnston SC (2009). Significance of large vessel intracranial occlusion causing acute ischemic stroke and TIA. Stroke.

[3] Yamauchi H, Higashi T, Kagawa S, Kishibe Y, Takahashi M (2013). Chronic hemodynamic compromise and cerebral ischemic events in asymptomatic or remote symptomatic large-artery intracranial occlusive disease. AJNR Am J Neuroradiol.

[4] Kuroda S, Houkin K, Kamiyama H, Mitsumori K, Iwasaki Y, Abe H (2001). Long-term prognosis of medically treated patients with internal carotid or middle cerebral artery occlusion: can acetazolamide test predict it?. Stroke.

[5] Group EIBS (1985). Failure of extracranial-intracranial arterial bypass to reduce the risk of ischemic stroke. Results of an international randomized trial. N Engl J Med.

[6] Powers WJ, Clarke WR, Grubb RL, Videen TO, Adams HP, Derdeyn CP (2011). Extracranial-intracranial bypass surgery for stroke prevention in hemodynamic cerebral ischemia: the carotid occlusion surgery study randomized trial. JAMA.

[7] Yu Jia Z, Sun Song Y, Jon Sheen J, Goo Kim J, Hee Lee D (2019). Endovascular recanalization of symptomatic non-acute intracranial artery occlusion: procedural and mid-term clinical outcomes in the anterior circulation. Interv Neuroradiol.

[8] Ma L, Liu YH, Feng H, Xu JC, Yan S, Han HJ (2019). Endovascular recanalization for symptomatic subacute and chronic intracranial large artery occlusion of the anterior circulation: initial experience and technical considerations. Neuroradiology.

[9] Aghaebrahim A, Jovin T, Jadhav AP, Noorian A, Gupta R, Nogueira RG (2014). Endovascular recanalization of complete subacute to chronic atherosclerotic occlusions of intracranial arteries. J Neurointerv Surg.

[10] Zheng M, Song Y, Zhang J, Zhao W, Sun L, Yin H (2019). Endovascular recanalization of non-acute symptomatic middle cerebral artery total occlusion and its short-term outcomes. Front Neurol.

[11] Yao YD, Liu AF, Qiu HC, Zhou J, Li C, Wang Q (2019). Outcomes of late endovascular recanalization for symptomatic non-acute atherosclerotic intracranial large artery occlusion. Clin Neurol Neurosurg.

[12] Gao P, Wang Y, Ma Y, Yang Q, Song H, Chen Y (2018). Endovascular recanalization for chronic symptomatic intracranial vertebral artery total occlusion: experience of a single center and review of literature. J Neuroradiol.

[13] Zhao W, Zhang J, Meng Y, Zhang Y, Zhang J, Song Y (2020). Symptomatic atherosclerotic non-acute intracranial vertebral artery total occlusion: clinical features, imaging characteristics, endovascular recanalization, and follow-up outcomes. Front Neurol.

[14] Dashti SR, Park MS, Stiefel MF, McDougall CG, Albuquerque FC (2010). Endovascular recanalization of the subacute to chronically occluded basilar artery: initial experience and technical considerations. Neurosurgery.

[15] He Y, Wang Z, Li T, Jiang WJ, Zhu L, Xue J (2013). Preliminary findings of recanalization and stenting for symptomatic vertebrobasilar artery occlusion lasting more than 24 h: a retrospective analysis of 21 cases. Eur J Radiol.

[16] Kang K, Yang B, Gong X, Chen X, Gu W, Ma G (2020). Cerebral hemodynamic changes after endovascular recanalization of symptomatic chronic intracranial artery occlusion. Front Neurol.

[17] He Y, Bai W, Li T, Xue J, Wang Z, Zhu L (2014). Perioperative complications of recanalization and stenting for symptomatic nonacute vertebrobasilar artery occlusion. Ann Vasc Surg.

[18] Gao F, Guo X, Han J, Sun X, Zhou Z, Miao Z (2021). Endovascular recanalization for symptomatic non-acute middle cerebral artery occlusion: proposal of a new angiographic classification. J Neurointerv Surg.

[19] Gao F, Han J, Guo X, Sun X, Ma N, Miao Z (2022). Endovascular recanalization for non-acute basilar artery occlusions with progressive or recurrent ischemic symptoms: a multicenter clinical experience. J Neurointerv Surg.

[20] Gao F, Guo X, Sun X, Liu Y, Wu Y, Miao Z (2021). Dual-roadmap guidance for endovascular recanalization of medically refractory non-acute intracranial arterial occlusions: consecutive multicenter series and technical review. J Neurointerv Surg.

[21] Gao F, Sun X, Guo X, Li D, Xu GD, Miao ZR (2021). Endovascular recanalization of symptomatic nonacute Intracranial Internal Carotid artery occlusion: proposal of a New Angiographic classification. AJNR Am J Neuroradiol.

[22] Hou Z, Yan L, Zhang Z, Jing J, Lyu J, Hui FK et al. High-resolution magnetic resonance vessel wall imaging-guided endovascular recanalization for nonacute intracranial artery occlusion. J Neurosurg. 2021:1–7. 10.3171/2021.9.JNS211770 34861645

[23] Gao F, Sun X, Zhang H, Ma N, Mo D, Miao Z (2020). Endovascular recanalization for Nonacute Intracranial vertebral artery occlusion according to a new classification. Stroke.

[24] Chao L, Qingbin M, Haowen X, Shanshan X, Qichang F, Zhen C (2021). Imaging predictors for endovascular recanalization of non-acute occlusion of Internal Carotid Artery based on 3D T1-SPACE MRI and DSA. Front Neurol.

[25] Chen YH, Leong WS, Lin MS, Huang CC, Hung CS, Li HY (2016). Predictors for successful endovascular intervention in chronic carotid artery total occlusion. JACC Cardiovasc Interv.

[26] Yang B, Kang K, Gao F, Mo D, Tong X, Song L et al. Association of occlusion time with successful endovascular recanalization in patients with symptomatic chronic intracranial total occlusion. J Neurosurg. 2022:1–10. 10.3171/2021.12.JNS212337 35120327

[27] Bouthillier A, van Loveren HR, Keller JT (1996). Segments of the internal carotid artery: a new classification. Neurosurgery.

[28] Seners P, Baron JC (2018). Revisiting ‘progressive stroke’: incidence, predictors, pathophysiology, and management of unexplained early neurological deterioration following acute ischemic stroke. J Neurol.

[29] Higashida RT, Furlan AJ, Roberts H, Tomsick T, Connors B, Barr J (2003). Trial design and reporting standards for intra-arterial cerebral thrombolysis for acute ischemic stroke. Stroke.

[30] Sun X, Tong X, Lo WT, Mo D, Gao F, Ma N (2017). Risk factors of subacute thrombosis after intracranial stenting for symptomatic intracranial arterial stenosis. Stroke.

[31] Furie KL, Kasner SE, Adams RJ, Albers GW, Bush RL, Fagan SC (2011). Guidelines for the prevention of stroke in patients with stroke or transient ischemic attack: a guideline for healthcare professionals from the american heart association/american stroke association. Stroke.

[32] Chimowitz MI, Lynn MJ, Derdeyn CP, Turan TN, Fiorella D, Lane BF (2011). Stenting versus aggressive medical therapy for intracranial arterial stenosis. N Engl J Med.

[33] Morino Y, Abe M, Morimoto T, Kimura T, Hayashi Y, Muramatsu T (2011). Predicting successful guidewire crossing through chronic total occlusion of native coronary lesions within 30 minutes: the J-CTO (Multicenter CTO Registry in Japan) score as a difficulty grading and time assessment tool. JACC Cardiovasc Interv.

[34] Christoforidis GA, Mohammad Y, Avutu B, Tejada A, Slivka AP (2006). Arteriographic demonstration of slow antegrade opacification distal to a cerebrovascular thromboembolic occlusion site as a favorable indicator for intra-arterial thrombolysis. AJNR Am J Neuroradiol.

[35] Puma JA, Sketch MH, Tcheng JE, Harrington RA, Phillips HR, Stack RS (1995). Percutaneous revascularization of chronic coronary occlusions: an overview. J Am Coll Cardiol.

[36] Li M, Zhang J, Pan J, Lu Z (2013). Obstructive coronary artery disease: reverse attenuation gradient sign at CT indicates distal retrograde flow–a useful sign for differentiating chronic total occlusion from subtotal occlusion. Radiology.

[37] Tan KH, Sulke N, Taub NA, Watts E, Karani S, Sowton E (1993). Determinants of success of coronary angioplasty in patients with a chronic total occlusion: a multiple logistic regression model to improve selection of patients. Br Heart J.

[38] Salarifar M, Mousavi MR, Saroukhani S, Nematipour E, Kassaian SE, Alidoosti M (2014). Percutaneous coronary intervention to treat chronic total occlusion: predictors of technical success and one-year clinical outcome. Tex Heart Inst J.

[39] Oktaviono YH, Rizal A, Al-Farabi MJ, Maghfirah I, Rachmi DA (2020). Coronary angiography characteristics as predictor of successful chronic total occlusion recanalization. Int J Angiol.

[40] Namazi MH, Serati AR, Vakili H, Safi M, Parsa SAP, Saadat H (2017). A novel risk score in Predicting failure or success for Antegrade Approach to Percutaneous Coronary intervention of chronic total occlusion: Antegrade CTO score. Int J Angiol.

[41] Alessandrino G, Chevalier B, Lefevre T, Sanguineti F, Garot P, Unterseeh T (2015). A clinical and angiographic Scoring System to predict the probability of successful first-attempt percutaneous coronary intervention in patients with total chronic coronary occlusion. JACC Cardiovasc Interv.

[42] Wang N, Fulcher J, Abeysuriya N, Adams M, Lal S (2018). Predictors of successful chronic total occlusion percutaneous coronary interventions: a systematic review and meta-analysis. Heart.

[43] Katsuragawa M, Fujiwara H, Miyamae M, Sasayama S (1993). Histologic studies in percutaneous transluminal coronary angioplasty for chronic total occlusion: comparison of tapering and abrupt types of occlusion and short and long occluded segments. J Am Coll Cardiol.

[44] Barger AC, Beeuwkes R 3rd, Lainey LL, Silverman KJ. Hypothesis: vasa vasorum and neovascularization of human coronary arteries. A possible role in the pathophysiology of atherosclerosis. N Engl J Med. 1984;310(3):175–7. 10.1056/NEJM198401193100307 6197652

[45] Liang W, Wang Y, Du Z, Mang J, Wang J (2021). Intraprocedural angiographic signs observed during endovascular thrombectomy in patients with Acute ischemic stroke: a systematic review. Neurology.

